# Effect of PVA Blending on Structural and Ion Transport Properties of CS:AgNt-Based Polymer Electrolyte Membrane

**DOI:** 10.3390/polym9110622

**Published:** 2017-11-15

**Authors:** Shujahadeen B. Aziz, Omed Gh. Abdullah, Sarkawt A. Hussein, Hameed M. Ahmed

**Affiliations:** 1 Advanced Polymeric Materials Research Lab., Department of Physics, College of Science, University of Sulaimani, Qlyasan Street, Sulaimani 46001, KRG, Iraq; omed.abdullah@univsul.edu.iq (O.G.A.); sarkawt.hussen@univsul.edu.iq (S.A.H.); hameed.ahmad@univsul.edu.iq (H.M.A.); 2 Development Center for Research and Training (DCRT), University of Human Development, Qrga Street, Sulaimani 46001, KRG, Iraq

**Keywords:** polymer blends, silver salt, dielectric constant, relaxation processes, Arrhenius model, AC conductivity

## Abstract

In this work, the role of poly(vinyl alcohol) (PVA) blending on structural and electrical properties of chitosan:silver nitrate systems is studied. The X-ray diffraction (XRD) results show that the crystalline phase of chitosan (CS) is greatly scarified by silver nitrate (AgNt) salt. The crystalline domain of CS:AgNt is more broadened at 10 wt % of PVA. The spike and semicircular arcs can be separated in impedance plots. At high temperatures, the spike regions remained. The direct current (DC) conductivity was calculated from the bulk resistance obtained from the impedance plots. The dielectric constant and DC conductivity versus PVA content exhibited similar behavior. The maximum DC conductivity at ambient temperature was 1.1 × 10^−6^ S/cm for 10 wt % of PVA. The DC ionic conductivity increased to 9.95 × 10^−5^ S/cm at 80 °C. Above 10 wt % of PVA, the drop in DC conductivity and dielectric constant were observed due to the increase in viscosity. Shifting of relaxation peaks towards the lower frequency revealed the increase of resistivity of the samples. The linear increase of DC conductivity versus 1000/T indicated that ion transport followed the Arrhenius model. The incomplete semicircular arc in Argand plots indicated the non-Debye type of relaxation process. The Argand plots were used to distinguish between conductivity relaxation and viscoelastic relaxation. Three regions were distinguished in the alternating current (AC) spectra of the blend electrolyte samples. The plateau region in AC spectra was used to estimate the DC conductivity. The estimated DC conductivity from the AC spectra was close to those calculated from the impedance plots.

## 1. Introduction

Recently, polymeric electrolytes (PEs) have attracted the attention of many researchers due to their diverse application in display technology and electrochromical devices [1]. PEs signifies an interesting group of ion conducting compounds, in which ion transport occurs through the solid membranes [2]. Since the original studies of Wright et al. and Armand et al. [1], solid polymer electrolytes have been broadly investigated. Polymer electrolytes are formed when low dissociation energy alkali metal salts are dissolved in polar polymers, and they have wide applications in batteries/fuel cells, electrochemical display devices/smart windows, and photoelectrochemical cells [3]. Recent study reveals that natural polymers are attractive for the synthesis of polymer electrolytes because they are environmentally friendly material and they have a natural tendency for degradation compared to synthetic polymers [4]. On the other hand, synthetic polymer materials made from petroleum sources are considered non-degradable because they produce toxic waste that causes to our environment [5]. Chitosan (CS) is a natural polymer that is produced from chitin by deacetylation with an alkali. Properties such as its biocompatibility, non-toxicity, and thermal stability up to 200 °C make CS potential polymer for the preparation of polymer electrolytes [6,7]. The presence of an amino (NH_2_) group and hydroxyl (OH) groups on the CS backbone structure is enough to dissolve inorganic salts [8]. The semi-crystalline polyvinyl alcohol, PVA, is an attractive material for use as a host polymer for electrolyte preparation and it has previously been employed in Zn-air batteries, rechargeable Ni-MH batteries, and direct methanol fuel cells [9]. Poly(vinyl alcohol) (PVA) is a synthetic polymer with a high dielectric constant and an excellent film-forming capacity. PVA is a hydrophilic polymer and has a high density of reactive chemical functional groups [10]. These functional (-OH) groups are significant for polymer blending with CS polymer. Recent studies revealed that it is possible to create new polymeric compounds with good chemical and physical properties through the blending of two polymers [11]. The blending between two polymers occurs as a result of interaction among the polar groups of the two blended polymers through intramolecular and intermolecular hydrogen bonding, that is, the blending takes place on the molecular level [12]. Earlier studies confirmed that ion transport and ionic mobility can be increased through the blending of two polymers [11]. Ion relaxation and charge transport mechanisms are the most intensively researched subjects in solid polymer electrolytes as a branch of condensed matter physics [13]. In our previous work [14], we showed that the blending of PVA with chitosan:AgNt systems plays a great role in tuning the surface plasmon resonance (SPR) peaks of reduced silver nanoparticles. This work aims to study the conductivity and relaxation processes associated with ion movement using AC impedance spectroscopy, which is an important technique used to examine the electrical and dielectric properties of materials. Thus, the main objective of this work is to study the relaxation processes and ion transport mechanism in silver ion-conducting polymer blend electrolyte membranes based on chitosan. The noticeable results obtained in the present work nominate this system to be used as a good polymer electrolyte membrane for gas separation, as most polymer membranes containing silver ions used for gas separation exhibit a highly amorphous structure.

## 2. Experimental Methods

### 2.1. Materials and Sample Preparation

**Figure 1:**
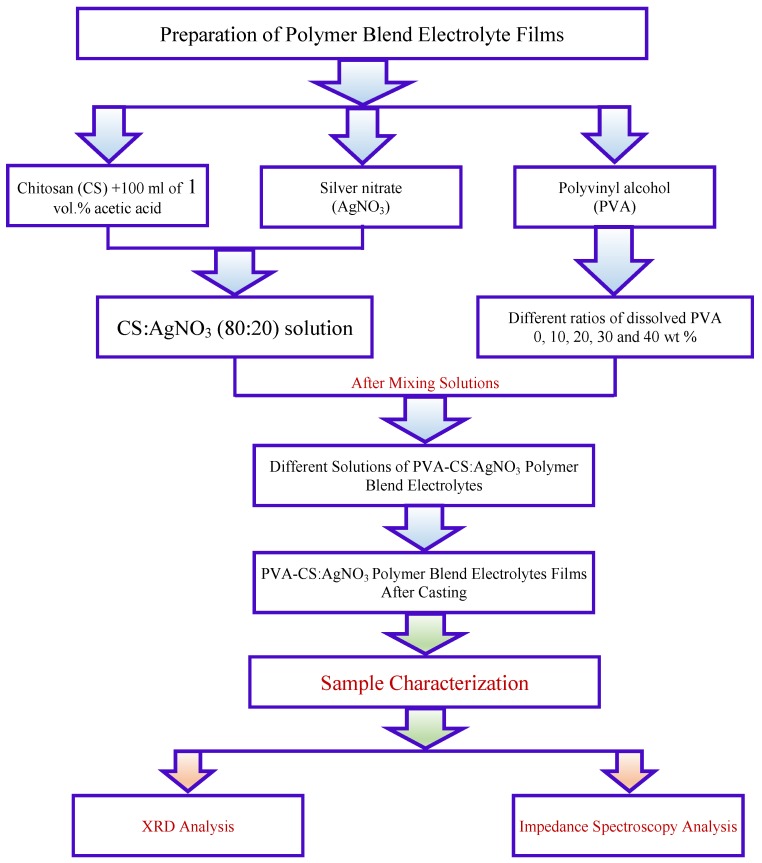
Flow chart for samples preparation and characterization techniques.

Ion conducting membranes were prepared by the solution cast method. The fixed ratio (80:20) of chitosan (CS) and silver nitrate (AgNO_3_) were dissolved in 100 mL of 1 vol % acetic acid. The solution was stirred for 24 h to achieve a uniform solution. The degree of deacetylation of CS is ≥75%. Different ratios of PVA of 98–99% hydrolyzed (molecular weight, *M*_w_ = 98,000 g/mol) were dissolved at 90 °C in distilled water separately under continuous stirring. The PVA solutions were left to cool down to ambient temperature and then were added separately to the CS:AgNt (80:20) solution under magnetic stirring to prepare polymer blend electrolytes. The polymer blend electrolyte samples were coded as CSPV0, CSPV1, CSPV2, CSPV3 and CSPV4 for CS:AgNt incorporated with 0, 10, 20, 30 and 40 wt % of PVA, respectively. The mixtures of these solutions were stirred for another 10 h. These solutions were transferred into plastic Petri dishes and left to dry at a temperature of 30 °C. The films were transported into a desiccator for further drying. The thickness of the films ranged from 123–125 μm. Figure 1 shows the flow chart for samples preparation and characterization techniques.

### 2.2. Structural and Electrical Characterization

The XRD was performed on the samples using an X-ray diffractometer (Bruker AXS, Billerica, MA, USA) with an operating voltage and current of 40 kV and 40 mA, respectively. The samples were scanned with a beam of monochromatic, X-radiation of wavelength λ = 1.5406 A^°^ and the glancing angles were in the range of 5° ≤ 2θ ≤ 80° with a step size of 0.1°. The electrical behavior of the electrolyte membranes were examined using a HIOKI 3531 Z Hi-tester within the frequency range of 50–1000 kHz.

## 3. Results and Discussion

### 3.1. Structural Analysis

**Figure 2:**
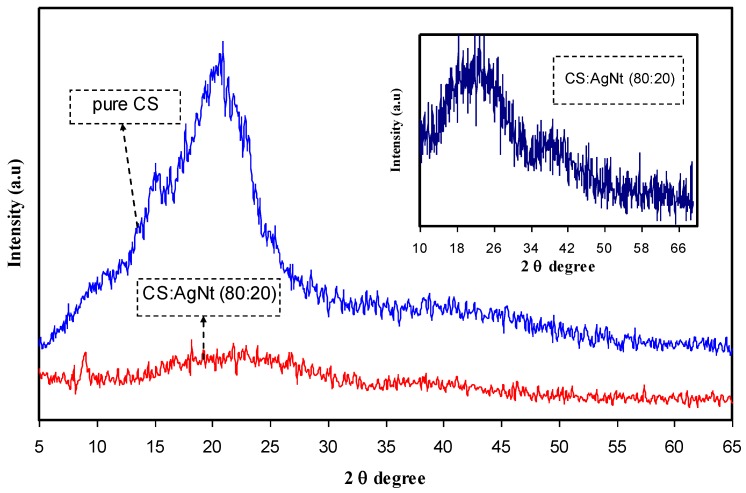
XRD pattern for pure CS and CS:AgNt samples.

**Figure 3:**
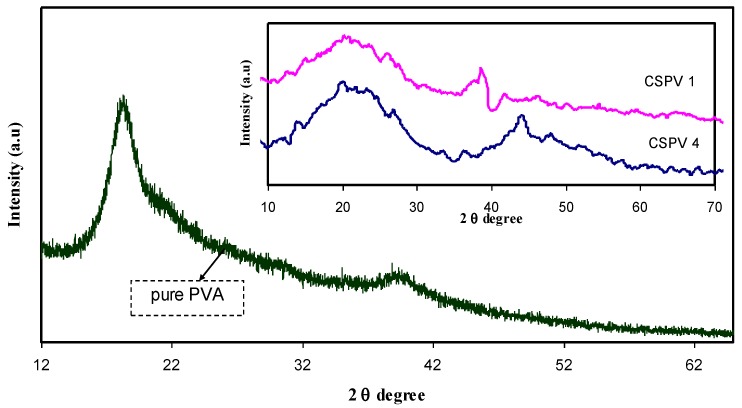
XRD pattern for pure PVA and blend samples.

Figure 2 shows the diffractogram of pure CS and CS doped with 20 wt % AgNt. It is clear that pure chitosan exhibits some peaks at 2θ = 15.6°, 17.4° and 21.8°. These peaks are usually characterized as a semi-crystalline polymer. The inset of Figure 1 shows that the main peak of chitosan, which appeared at 2θ = 21.8°, almost scarified and changed to a broad peak. Earlier study verified that the lowering of intensity and enlargement of the XRD pattern indicates an increase in amorphous phase [8]. The occurrence of molecular interactions between the functional groups of the polymer and cations of the doping salt hinder the ordering of the crystalline phase and consequently induce an increase in amorphous fraction [3]. Figure 3 shows the XRD pattern of pure PVA. The inset of Figure 3 shows the XRD pattern for CS:AgNt incorporated with 10 wt % PVA (CSPV1) and 40 wt % PVA (CSPV4). It can be seen that the XRD pattern of pure PVA showed a peak around 20° corresponding to the semi-crystalline nature of pure PVA [10]. The existence of OH groups along the main chain of PVA is enough to provide strong intermolecular and intramolecular hydrogen bonding in PVA. The broad peak at 2θ = 40.7° can be ascribed to amorphous phases in PVA. The inset of Figure 2 shows that at 10 wt % PVA (CSPV1), the system is more amorphous and the peak at 2θ = 21.1° is more broadened compared to that of the CSPV4 system. This indicates that the reduction in crystalline phase may results from the blending of CS and PVA at 10 wt % PVA. The addition of more PVA means the incorporation of more OH groups, and this enhances intermolecular and intramolecular hydrogen bonding through the polymer blending between CS and PVA, and thus may develop the crystalline phase again as observed in the CSPV4 system. The disappearance of the main peak of PVA inside the CS:AgNt system indicates a good miscibility between the CS and PVA polymers.

### 3.2. Impedance Analysis

**Figure 4:**
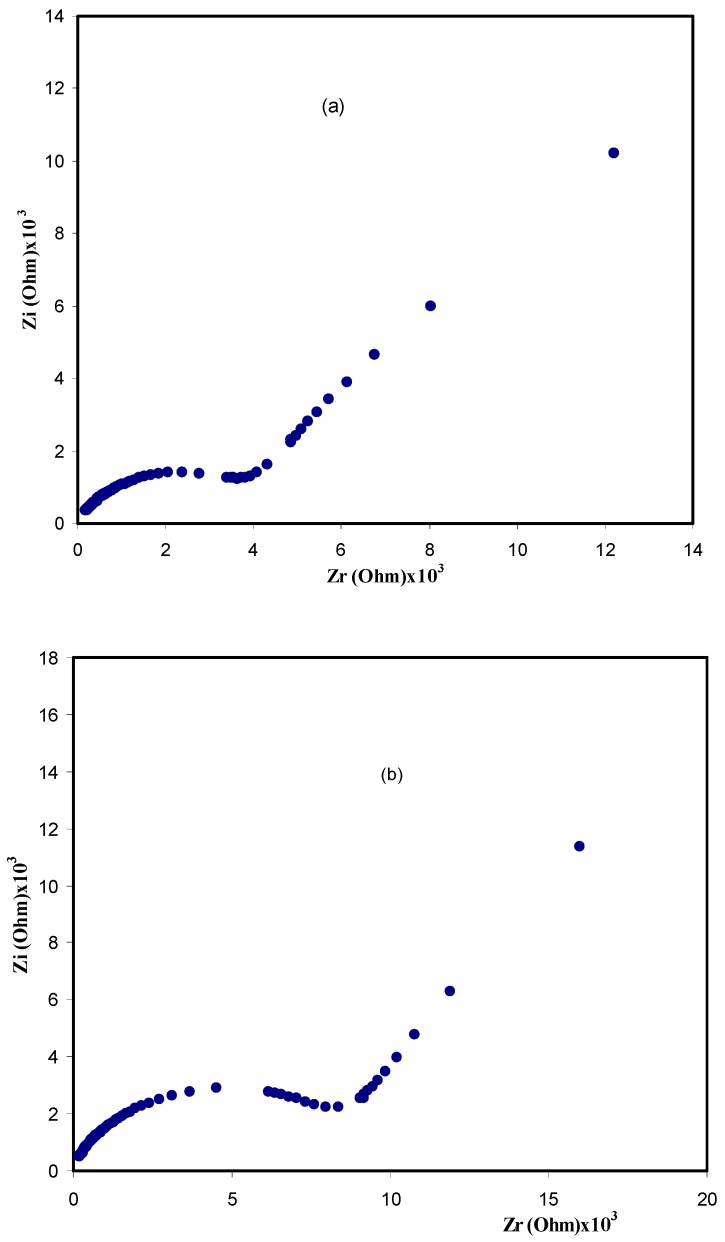

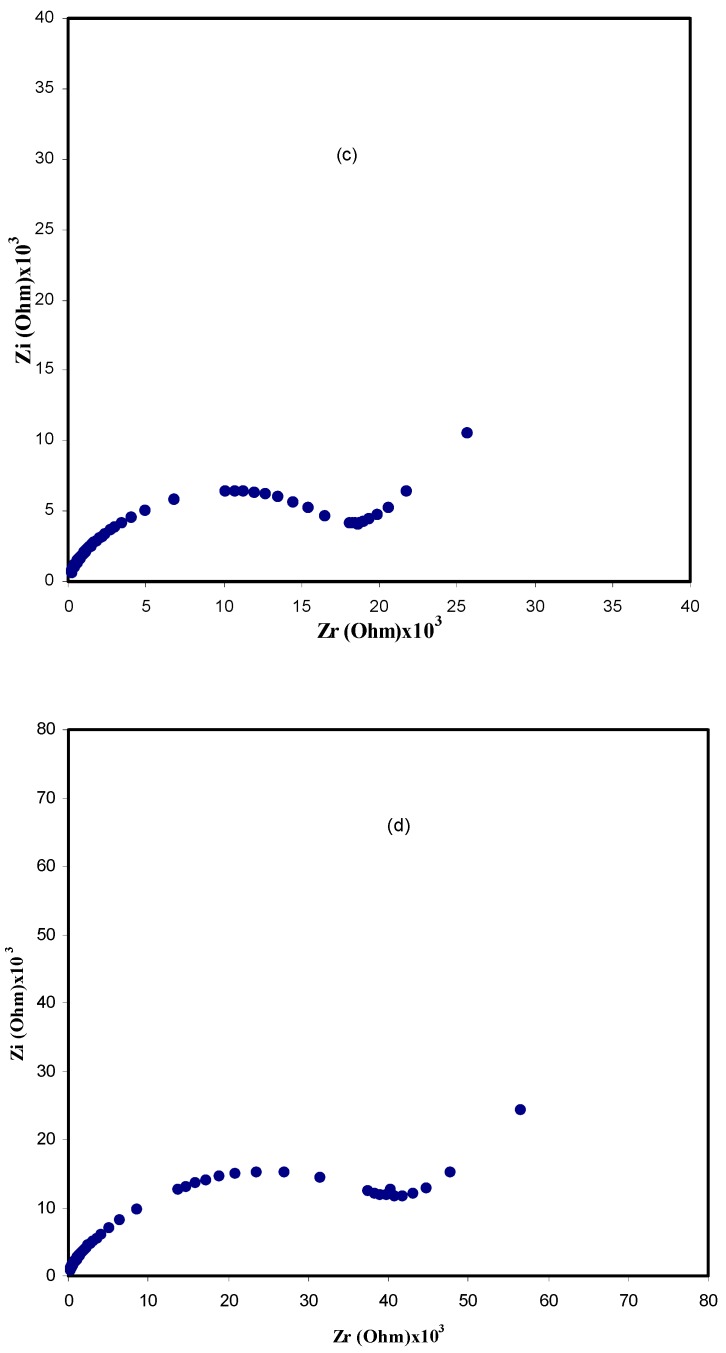
Impedance plots for (**a**) CSPV 1; (**b**) CSPV 2; (**c**) CSPV 3; and (**d**) CSPV4 sample.

**Figure 5:**
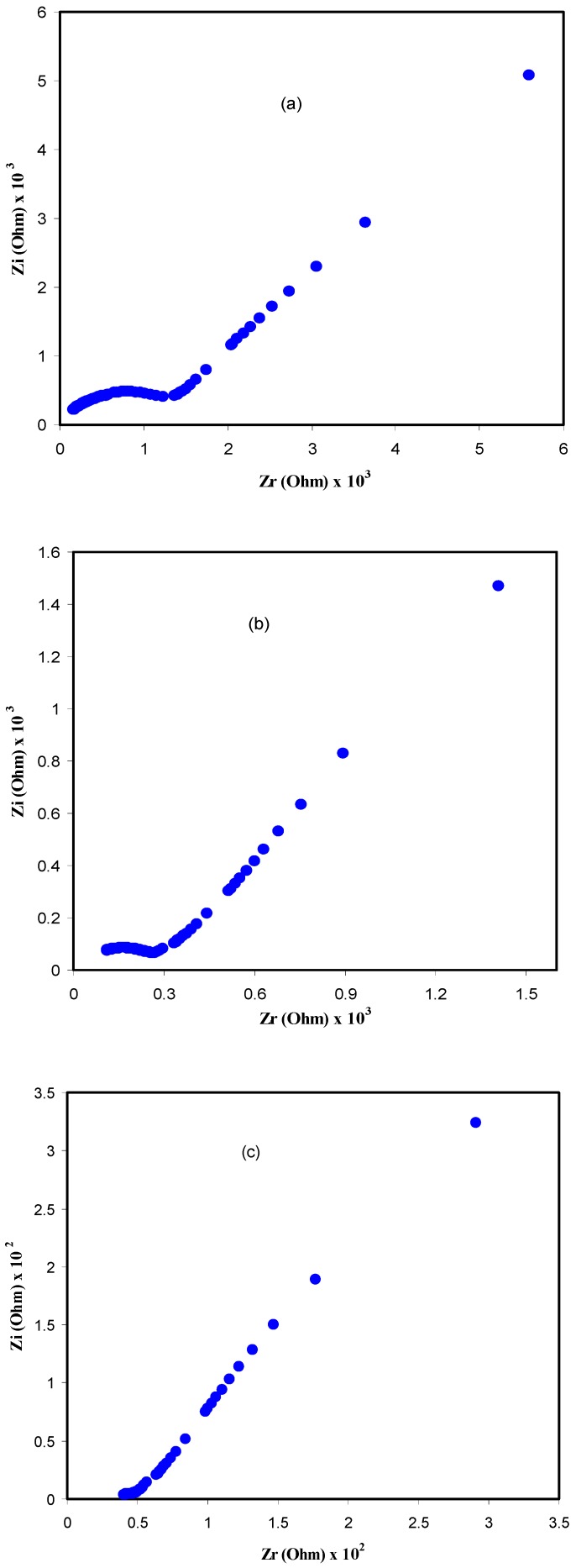
Impedance plots for the CSPV1 sample at (**a**) 50 °C, (**b**) 70 °C and (**c**) 90 °C.

Electrochemical impedance spectroscopy (EIS) is a common technique used to study charging and transport phenomena in ion-conducting and conjugated polymers [15]. Figure 4 shows the Nyquist plot for all of the samples. Clearly, the impedance plot of the samples comprises a broadened semicircle in the high frequency region followed by a tail (spike) in the lower frequency region. The incomplete semicircle at higher frequencies is attributed to the bulk resistance of the samples, whereas the data points at low frequencies can be ascribed to the formation of double layer capacitance at the electrode/sample interface [16,17]. Blocking electrodes such as stainless steel are usually used to study the electrical properties of ion-conducting polymer membranes. In the case of using ion-conducting electrodes, it is impossible to obtain any impedance plots and thus the bulk resistances cannot be estimated. To gain further insight, the impedance plots at different temperatures are presented for the CSPV1 sample in Figure 5. The disappearance of the semicircular portion at high temperatures in impedance plots reveals that the charge carriers are almost ions [18,19]. It is obvious that the bulk resistance of CSPV1 is smaller than the bulk resistance of CSPV4. This may be related to the fact that CSPV1 is more amorphous compared to CSPV4 (see the inset of Figure 3).

### 3.3. Conductivity and Dielectric Constant Study

**Figure 6:**
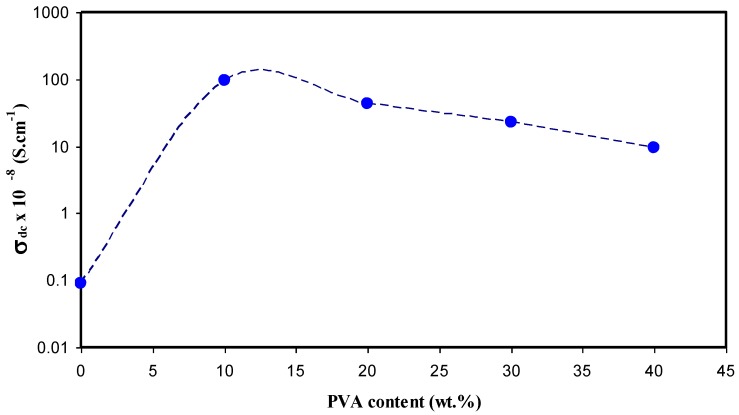
The DC conductivity versus PVA concentration.

It is well reported that the DC ionic conductivity of polymer ion-conducting electrolytes depends on the ion concentration and their mobility, as follows [20,21]:

*σ* = *Σ n_i_z_i_ µ_i_* (1)

where *n_i_*, *z_i_* and *µ_i_* refer to the number of charge carriers, the ionic charge, and the ionic mobility, respectively. From Equation (1), it is clear that the increase of DC conductivity is achieved either by the increase of carrier density or mobility. The ionic conductivity could be estimated from the bulk resistance (R_b_) obtained from the intersection of semicircular arcs with the real axis (*Z_r_*) using the relation *σ_dc_* = *l*/*RA*, where *l* is the electrolyte membrane thickness and *A* its area under study [22]. Figure 6 illustrates the DC conductivity versus PVA concentration. It is clear that above 10 wt % of PVA, the DC conductivity drops. The possible interpretation of this phenomena is that when more PVA is added to the CS:AgNt system, the viscosity may increase and thus the ion transport may decrease. The highest DC conductivity obtained in the present work was found to be 1.1 × 10^−6^ S/cm, for CS:AgNt blended with 10 wt % of PVA. The ionic conductivity obtained in the present study is comparable to those reported by other researchers. Shuhaimi et al. [23] reported a DC conductivity of about 2.10 × 10^−6^ S cm^−1^ for methylcellulose (MC)-doped with 25 wt % of ammonium nitrate (NH_4_NO_3_) at room temperature. It is also close to that recorded (2.29 × 10^−6^ S cm^−1^) for plasticized PMMA-LiCF_3_SO_3_ by Ali et al. [24], for 35 wt % of LiTf. The high DC conductivity of the present work at 20 wt % of AgNt compared to values reported in the literature may be related to the role of blending.

Previous studies confirmed that the study of the dielectric constant is crucial for detecting the conductivity behavior of polymer electrolytes and understanding the ion transport mechanism [25,26,27]. The real (*Z_r_*) and imaginary (*Z_i_*) part of complex impedance (Z*) was used for the evaluation of the dielectric constant using the following equation [28]:

(2) $$\varepsilon^{\prime }=\frac{Z_{i}}{\omega C_{o}(Z_{r}{}^{2}+Z_{i}{}^{2})}$$

**Figure 7:**
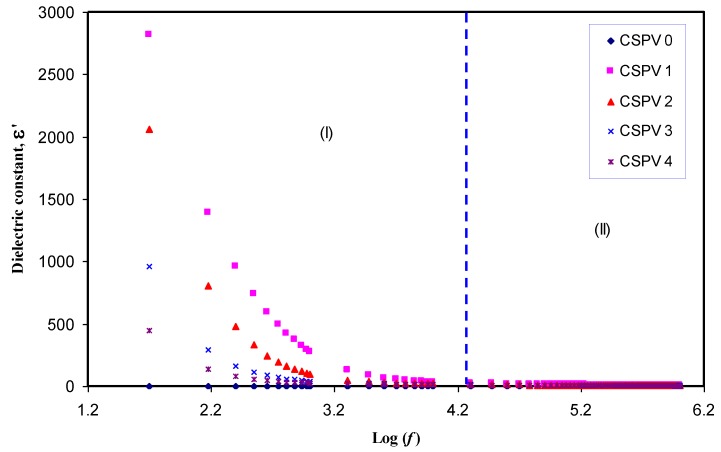
Dielectric constant as a function of frequency for all of the samples.

**Figure 8:**
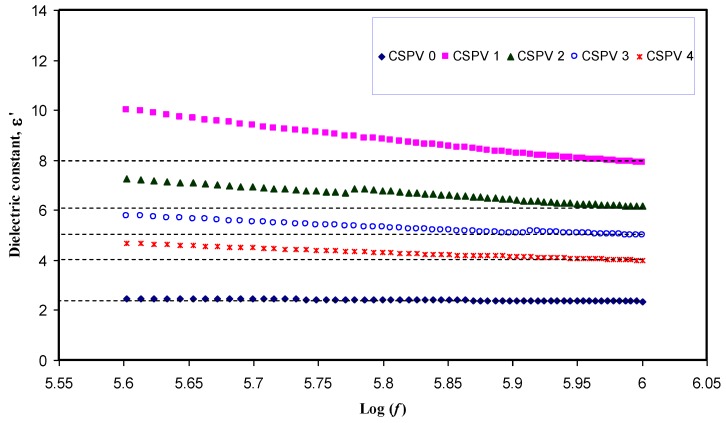
High frequency region of the dielectric constant as a function of frequency.

**Figure 9:**
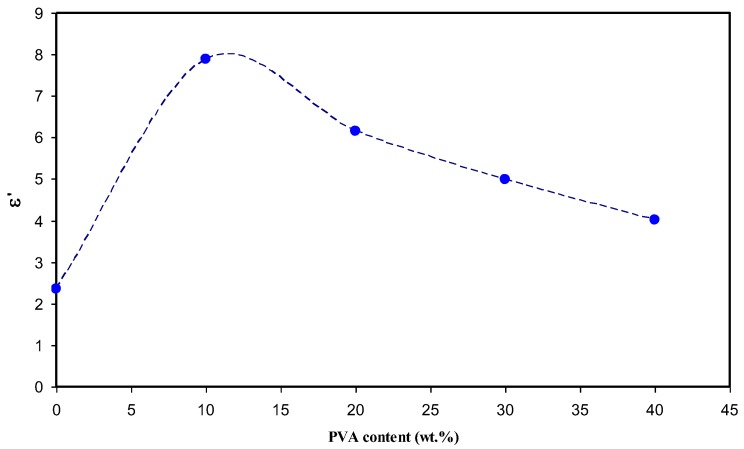
Dielectric constant as a function of PVA concentration.

Here, *C_o_* is the vacuum capacitance given by *ε_o_a/t*, where *ε_o_* is the permittivity of free space and is equal to 8.85 × 10^−12^ F/m. The angular frequency ω is equal to *ω* = 2*πf*, where *f* is the frequency of the applied field. Figure 7 shows the variation of dielectric constant for pure CS and all of the blend electrolyte samples. It is clear from Figure 7 that the value of the dielectric constant (ɛ′) is high at low frequencies. The large values of ɛ’ at low frequencies can be ascribed to the accumulation of a large amount of charges at the electrode-electrolyte interface [29]. It is obvious that the dielectric constant values begin to drop at high frequencies. This is because there is no excess ion diffusion, and the dipole molecules do not have sufficient time to orient themselves in the direction of the applied electric field at high frequencies [25,29]. The high frequency region of the dielectric constant is plotted separately and presented in Figure 8. It is clear that at 10 wt % of PVA, the dielectric constant reaches the maximum value and then decreases for other weight percentages of PVA. The increase in dielectric constant is important because the dissociation of more ions may take place that are able to participate in polarization as well as in conduction [25]. It is well known that increasing the percentage of PVA in the CS:AgNt electrolyte may increase the viscosity; each Ag^+^ cation may become surrounded by more OH functional groups, and thus ion movement may become more difficult in the viscous medium. Consequently, the DC conductivity and dielectric constant, which are both functions to ion movement and polarization, will drop. The behavior of dielectric constant versus PVA content is shown in Figure 9. It is obvious that dielectric constant versus PVA concentration has a similar trend to that observed for DC conductivity versus PVA content. The similar trends of dielectric constant and DC conductivity versus PVA content reveal that dielectric constant study is a powerful technique to elucidate the conductivity behavior of polymer electrolytes.

### 3.4. Temperature-Dependent Conductivity

**Figure 10:**
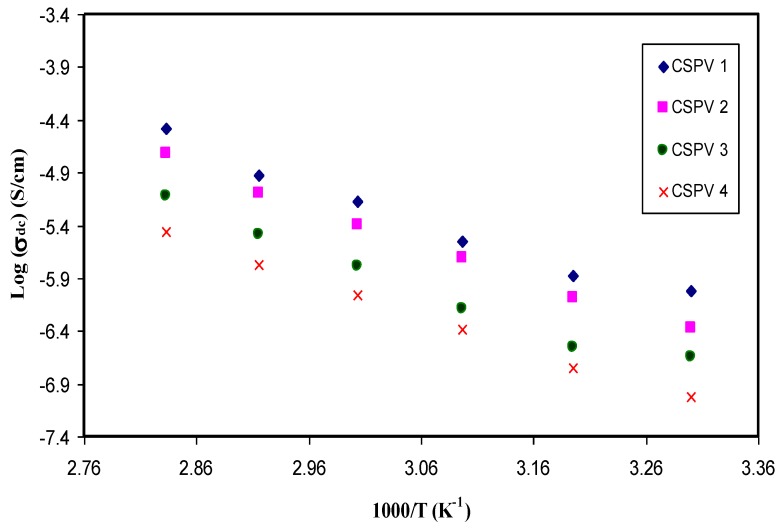
DC conductivity against 1000/T for blend electrolyte samples.

Figure 10 shows the variation of DC conductivity versus the reciprocal of temperature. The linear increase of conductivity with increasing temperature can be observed and can be explained by the Arrhenius model. The increase of DC conductivity at high temperatures can be ascribed to the re-dissociation of ions and thus the increase of the number of charge carriers [30]. It was reported that polymer chain motion at high temperatures as a result of the increment of free volume may help the ion mobility by providing more pathways through which ions can move [3,31]. The activation energy can be determined using the Arrhenius relation [32]:

(3) $$\sigma_{dc}=\sigma_{o}\exp^{\left(\frac{-E_{a}}{K_{B}T}\right)}$$

where *σ_o_* is the pre-exponential constant, which is related to the number of ion carriers in the membranes, *K_B_* is the Boltzmann constant, *T* is the absolute temperature, and *E_a_* is the energy of activation. The activation energy for the CSPV1 system was 0.65 eV, which increased to 0.68 eV for the CSPV4 system. The activation energies for blend electrolyte films were calculated from the slope of the straight line of Figure 10. The decrease in the electrical conductivity and increase in the activation energy may be related to the fact that the addition of larger amounts of PVA may increase the viscosity and thus impede ion transport.

### 3.5. Relaxation Study

The employ of an electric modulus can aid in identifying the bulk relaxation at low frequencies. Therefore, ordinary troubles like electrode polarization (EP), space charge injection phenomena, and conduction effects, which become visible to ambiguous relaxation in dielectric study, can be resolved in electric modulus formalism [7,33]. The real (*Z_r_*) and imaginary (*Z_i_*) parts of impedance (Z*) were also used for the estimation of electric modulus parameters using the following equations [7,19]:

(4) $$M^{\prime }=\omega C_{o}Z_{i}$$

(5) $$M^{\prime \prime }=\omega C_{o}Z_{r}$$

**Figure 11:**
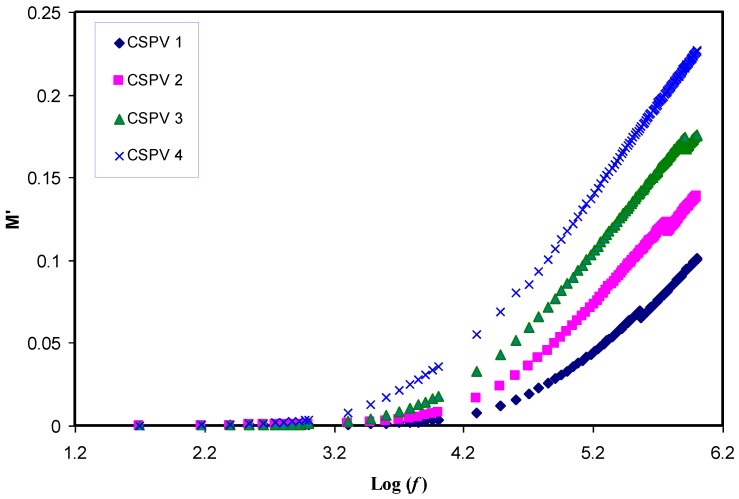
Real part of electric modulus (*M*′) versus frequency for all the blend electrolyte samples at room temperature.

**Figure 12:**
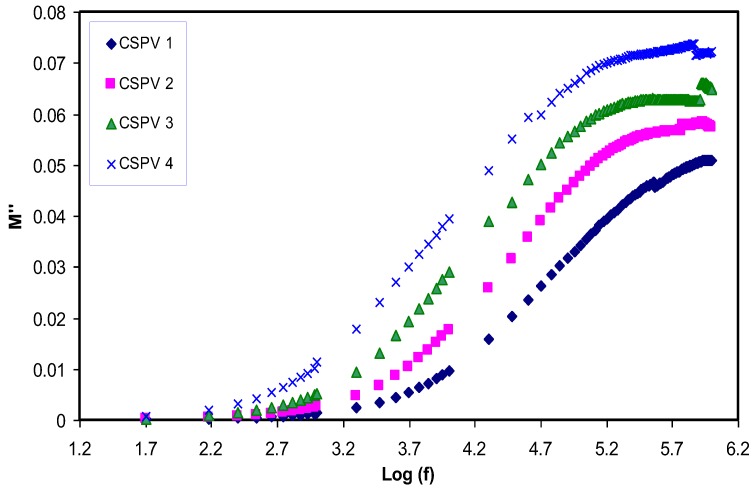
Imaginary part of electric modulus (*M*′′) versus frequency for blend electrolyte samples at room temperature.

**Figure 13:**
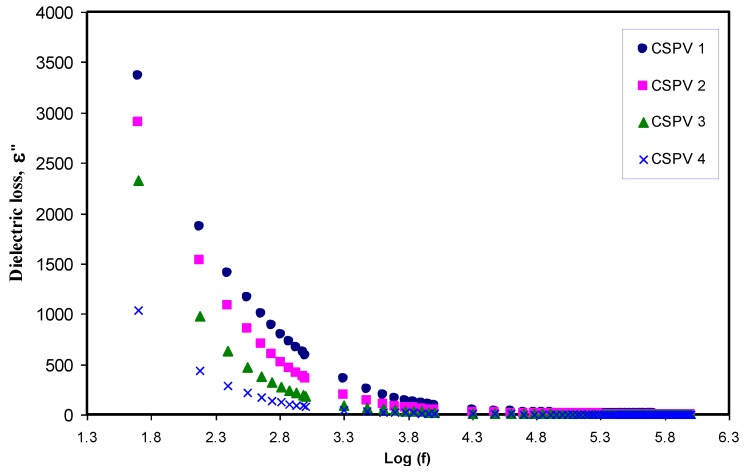
Dielectric losses versus frequency for blend electrolyte samples at room temperature.

Figure 11 shows that the *M*′ value approaches zero at low frequencies due to the high value of capacitance associated with double layer charges building up between the sample and electrodes [2]. Compared to the dielectric constant pattern, the *M*′ spectra exhibit a very different trend. The high value of the dielectric constant (see Figure 7) was observed at low frequencies. Because the electric modules (*M*′ and *M*′′) are reciprocal of a complex dielectric constant, they show a minimum value at high frequencies. The imaginary part of the modulus spectra (*M*′′) is presented in Figure 12. Recent reports in the literature have illustrated the interest of using *M*-formalism for analyzing electrical relaxation processes [34]. The unsymmetrical profile of *M*″ indicates that the simple exponential Debye is inappropriate to describe the relaxation. Conductivity relaxation peaks can be observed in Figure 12. The appearance of a relaxation peak in *M*′′ spectra with no peaks in the dielectric loss spectra (see Figure 13) carry some meaning from the physics point of view. The manifestation of peaks in *M*′′ spectra is evident due to the fact that ionic motion and polymer segmental motion are strongly coupled [35,36]. Clearly, the relaxation peaks shifted to towards the origin with increasing PVA concentration. This reveals that the relaxation time increases with the increase in PVA concentration. The increase of relaxation time is related to the decrease of ionic mobility [17]. In our previous works, we observed the decrease of relaxation times with increasing the conductivity [2,7,17,19,29], but in the present work the increase of relaxation time with decreasing conductivity was noted.

**Figure 14:**
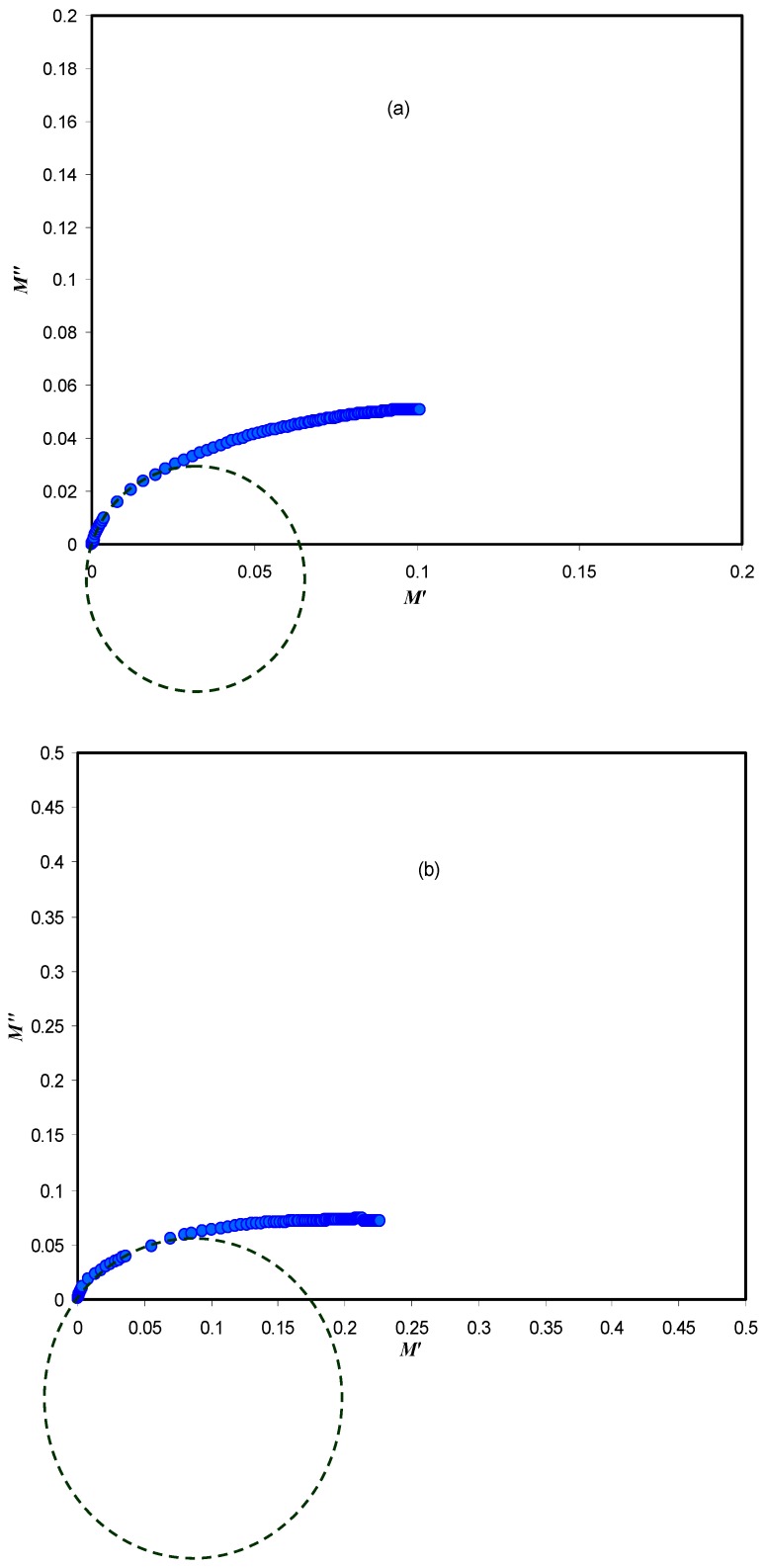
Argand plots for (**a**) CSPV1 and (**b**) CSPV4 samples.

The Argand plots for the CSPV1 and CSPV4 samples are shown in Figure 14. It can be seen that the Argand plots exhibit incomplete semicircular arcs. This indicates the non-Debye type relaxation process. The Debye model is developed for non-interacting identical dipoles [26,37]. Thus, the non-Debye behavior is due to the fact that in real material there is more than one type of polarization mechanisms and a many interactions between ions and dipoles. This results in a distribution of relaxation times. Moreover, it can be seen that with increasing PVA concentration, the *M*′′-*M*′ curve deviates more from the semicircular arc and the tail is depressed more towards the *M*′ axis due to the increase of resistivity. Depressed semicircles, such as those observed in the Argand plots, are a signature of deviation from ideal Debye behavior. With increasing the PVA concentration, the intersection of plots with the real axis moves away from the origin, indicating the decrease in mobility [38]. Also, with increasing the PVA concentration, the *Z_i_* and *Z_r_* values are increased and thus the *M*′′-*M*′ values ($M^{\prime \prime }=\omega C_{ο}Z_{r}$, $M^{\prime }=\omega C_{ο}Z_{i}$) move away from the origin. The study of Argand plots is crucial to distinguish between conductivity relaxation and viscoelastic relaxations processes. The conductivity relaxation matches the Debye model in which the diameter of the *M*′′-*M*′ curve coincides with the *M*′ axis, that is, the *M*″-*M*′ curve exhibits a complete semicircular arc and thus a single relaxation time can be estimated. On the other hand, if the *M*′′-*M*′ curve shows incomplete semicircular arcs, this means that there is a distribution of relaxation times. Consequently, ion transport occurs through the viscoelastic relaxations [39]. From Figure 14, it is clear that the diameters of the semicircles are well below the real axis, which reveals the distribution of relaxation times. Thus, ion transport occurs through the viscoelastic relaxation process.

**Figure 15:**
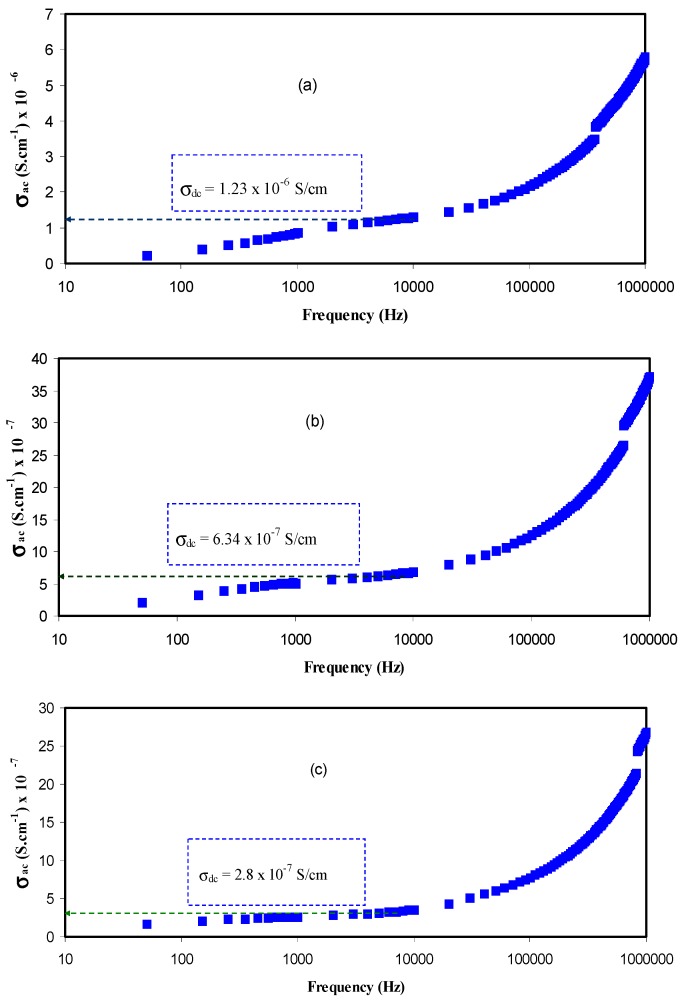

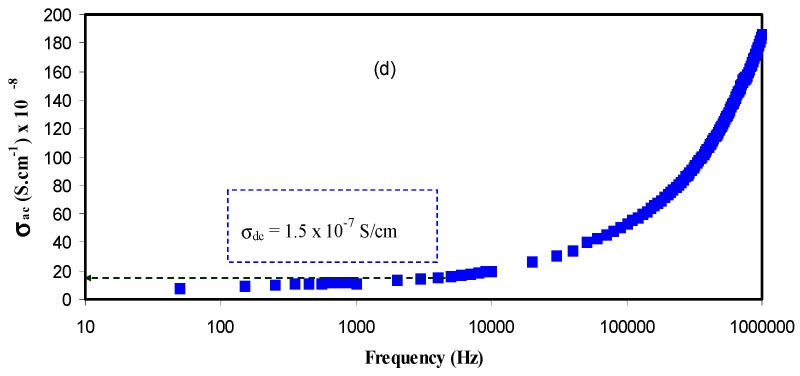
AC conductivity spectra for (**a**) CSPV 1; (**b**) CSPV2; (**c**) CSPV3; and (**d**) CSPV4 samples.

Previous studies established that determining AC conductivity versus frequency can be considered as a precise method to estimate the DC electrical conductivity [17,19,40]. This is related to the fact that a plateau region in AC spectra is almost frequency independent and can be used to estimate the DC conductivity [27,41]. Figure 15 shows the variation of AC conductivity (*σ_ac_*) with frequency for all of the blend electrolyte samples. The extension of the flat region of AC conductivity to the *Y*-axis can be used to estimate the DC conductivity. It is obvious that the flat region increases appreciably with increasing the PVA concentration, while the electrode polarization (EP) tail decreases [27,32]. The departure from the flat section in the AC conductivity spectrum may be attributable to the EP effect [42]. The achieved DC ionic conductivities for different PVA concentrations are presented as the inset inside the plots below.

## 4. Conclusions

The XRD results established that the crystalline region of chitosan (CS) is greatly scarified by silver nitrate (AgNt) salt. The crystalline domain of CS:AgNt is more broadened at 10 wt % of PVA and exhibits a high DC conductivity of about 1.1 × 10^−6^ S/cm. This is crucial for gas separation, as most polymer membranes containing silver ions used for gas separation exhibit a highly amorphous structure and should have high ion transport conductivity. The spike and semicircular arcs can be separated in impedance plots. The DC conductivity was calculated from the bulk resistance obtained from the impedance plots. At high temperatures, the spike regions remained, which indicates that the charge carriers are almost ions. The dielectric constant and DC conductivity versus PVA content exhibits similar behavior. The maximum DC conductivity at ambient temperature is 1.1 × 10^−6^ S/cm for 10 wt % of PVA. The DC ionic conductivity increased to 9.95 × 10^−5^ S/cm at 80 °C. Above 10 wt % of PVA, the drop in DC conductivity and the dielectric constant were observed due to the increase of viscosity. The shifting of relaxation peaks towards the lower frequency reveals the increase of resistivity of the samples. The linear increase of DC conductivity versus 1000/T indicates that ion transport follows the Arrhenius model. The appearance of peaks in *M*″ spectra with no peaks in *ε*″ spectra confirms the strong correlation between ion transport and polymer segmental motion. The Argand plots were used to distinguish between conductivity relaxation and viscoelastic relaxation processes. The incomplete semicircular arc in Argand plots indicates the non-Debye type of relaxation process. The diameters of the semicircles are well below the real axis, which reveals that ion transport occurs through the viscoelastic relaxation process. Three regions were distinguished in the AC spectra of the blend electrolyte samples. The plateau regions in AC spectra were used to estimate the DC conductivity. The estimated DC conductivity from the AC spectra was close to those calculated from the impedance plots.
